# Nuclear HER3 expression improves the prognostic stratification of patients with HER1 positive advanced laryngeal squamous cell carcinoma

**DOI:** 10.1186/s12967-021-03081-0

**Published:** 2021-09-27

**Authors:** Giovanni Almadori, Antonella Coli, Eugenio De Corso, Dario Antonio Mele, Stefano Settimi, Giovanni Di Cintio, Francesca Brigato, Domenico Scannone, Thomas E. Carey, Gaetano Paludetti, Libero Lauriola, Franco Oreste Ranelletti

**Affiliations:** 1 Unit of Head and Neck Oncology, “A. Gemelli” University Hospital Foundation IRCCS-Catholic University of the Sacred Heart, Largo A. Gemelli 8, 00168 Rome, Italy; 2 Unit of Otorhinolaryngology, “A. Gemelli” University Hospital Foundation IRCCS, Roma, Italy; 3 Università Cattolica del Sacro Cuore, Roma, Italy; 4 Unit of Anatomic Pathology, “A. Gemelli” University Hospital Foundation IRCCS, Roma, Italy; 5 Department of Otolaryngology/Head and Neck Surgery, University of Michigan, Ann Arbor, MI USA; 6 Department of Histology, Università Cattolica del Sacro Cuore, Roma, Italy

**Keywords:** Laryngeal squamous cell carcinoma, HER family receptors, HER phenotypes, HER1-HER3 co-expression, HER1, Nuclear HER3, Tumor differentiation, Prognostic role

## Abstract

**Background:**

Compared to the other members of human epidermal growth factor family receptors (HER), the role of HER3 has not been well defined in laryngeal cancer. The predictive and prognostic role of HER3 has been the focus of clinical attention but the research findings are contradictory, especially in laryngeal squamous cell carcinoma (LSCC). The variable localization of HER3 within cancer cells and the role of HER3 in primary and acquired resistance to HER1-targeted therapies remain unclear.

**Methods:**

We performed a retrospective analysis of two cohorts of 66 homogeneous consecutive untreated primary advanced LSCC patients, in which co-expression of HER1, HER2 and HER3 receptors was investigated by semi-quantitative immunohistochemistry. The association of their pattern of expression with survival was evaluated by Kaplan–Meier and Cox’s proportional hazard analyses. Multivariable Cox proportional hazards models were developed to predict median 2- and 3-year RFS and 2.5- and 5-year OS. The Akaike information criterion technique and backwards stepwise procedure were used for model selections. The performance of the final Cox models was assessed with respect to calibration and discrimination.

**Results:**

Immunohistochemical labeling for HER1 and HER2 was localized both in the cell membrane and in the cytoplasm, while HER3 labeling was observed both in the cell cytoplasm and in the nucleus. HER3 expression was inversely correlated with HER1 positivity. The expression patterns of HERs were associated with tumor differentiation. In both cohorts of patients, HER1 expression was associated with reduced relapse-free (RFS) and overall survival (OS). In HER1 positive tumors, the co-expression with nuclear HER3 was associated with better RFS and OS, compared with HER3 negative tumors or tumors expressing HER3 at cytoplasmic level. HER3 expressing tumors had a higher Geminin/MCM7 ratio than HER3 negative ones, regardless of HER1 co-expression. Multivariable analyses identified age at diagnosis, tumor site, HER1, HER3 and age at diagnosis, tumor stage, HER1, HER3, as covariates significantly associated with RFS and OS, respectively. Bootstrapping verified the good fitness of these models for predicting survivals and the optimism-corrected C-indices were 0.76 and 0.77 for RFS and OS, respectively.

**Conclusions:**

Nuclear HER3 expression was strongly associated with favourable prognosis and allows to improve the prognostic stratification of patients with HER1 positive advanced LSCC carcinoma.

**Supplementary Information:**

The online version contains supplementary material available at 10.1186/s12967-021-03081-0.

## Background

Laryngeal squamous cell carcinoma (LSCC) remains one of the most common cancers of the upper respiratory tract and occurs more commonly in men than in women, if compared to other head and neck cancers. In ~60% of patients, LSCC presents as a locally advanced disease at diagnosis, becoming one of the few tumors in which the 5-year survival rates have decreased over the past 40 years, even though the overall incidence is declining [1]. In the last two decades, the use of non-surgical larynx-preservation strategies, including radiotherapy (RT) with concurrent cisplatin, induction chemotherapy (CT) followed by RT or RT alone, increased as an alternative to total laryngectomy (TL). Thus, over time the decision-making changed from survival at all costs to survival with maximum functional outcomes, with a fine balancing treatment between overall survival, larynx function preservation, and quality of life. Unfortunately, these efforts have not met with improvements in overall survival (OS) rates, even though preservation of laryngeal function can be achieved in > 50% of patients, with a long laryngectomy-free survival. This highlights the need for further translational research and innovation in the field of molecular medicine, hoping that the identification of new biomarkers could be useful in the development of precision medicine and personalized treatment, thereby improving the oncological result and reducing the corresponding toxicity.

Targeted therapies are at the forefront of personalized medicine in LSCC. Epidermal growth factor receptor (EGFR/HER1) inhibition reprents a rational strategy focusing on molecular targets and the anti-EGFR monoclonal antibody cetuximab has been increasingly used in combination with RT, since its approval in 2006 [2]. Moreover, in comparison with conventional RT, bioradiotherapy (BioRT) with cetuximab significantly improves locoregional control rates and OS without any increase in unmanageable toxicity [3]. However, the results of different clinical trials on the subject are not conclusive and more investigations are necessary to clarify HER1 biology and patient bioselection [3, 4]. In fact, intrinsic and acquired resistance during BioRT with cetuximab inevitably occurs and various mechanisms for resistance to cetuximab have been suggested, including constitutive activation of HER1-mediating signaling molecules and/or activation of alternative pathways [5]. Furthermore, the sensitivity to cetuximab of the tumor cells cannot be accurately predicted by only HER1 expression.

Members of the HER family receptor tyrosine kinases and their respective ligands constitute a robust biologic system that plays a key role in regulation in cell proliferation, survival, and differentiation. In a previous retrospective study [6], we investigated the potential prognostic value of each HER family member receptor in patients with LSCC, receiving upfront surgery and postoperative RT. We found that HER1 expression was directly associated with the risk of relapse and death, while HER2, HER3 and HER4 expression was inversely associated.

HER dimerization is required for signal transduction to occur through formation of homodimeric or heterodimeric kinase-active complexes which differ functionally and represent essential information for the development of predictive biomarkers to be used in clinical trials [7]. In this respect, it has been reported that, in head and neck squamous cell carcinoma (HNSCC), the analysis of combined expression of HER family members improve the predicting power over that for any individual member [6, 8].

Recently, the role of HER3 in primary and acquired resistence to HER1-targeted or other targeted therapies in various cancers has attracted considerable attention. Respect to the other HER family receptor members, the role of HER3-mediated pathways has not been well defined in HNSCC, more particularly in LSCC. Some studies in HNSCC have shown that tumors with HER3 overexpression, mainly localized at the cytoplasmic membrane, have a poorer prognosis [8–11], while de Vicente et al. [12] did not report any correlation between survival and HER3 expression. Because of these contradictory findings, HER3 expression is not yet considered to be a potential prognostic indicator for anti-HER3 targeting in clinical practice, at least in the LSCC.

It is known that minichromosome maintenance (MCM) proteins mark all non-quiescent cells, whereas geminin identifies the proportion of actively proliferating cells that have entered S-phase, but not exited mitosis [13]. High levels of geminin expression promote G1 to S progression and accumulation of cancer cells in the S-G2-M phase of the cell cycle, where they are most chemo-radio sensitive [14]. Geminin expression, as a predictive marker of response to chemoradiotherapy (CRT), complements the prognostic informations obtained from MCM7 expression. In particular, Geminin/MCM7 ratio behaves as a reliable independent prognostic marker of the clinical outcome in LSCC patients [15]. At this purpose, we evaluated the association between expression pattern of HER family members and the ratio of Geminin/MCM7 labeling index as an estimate of the fraction of cancer cells in the S-G2-M phase of the cell cycle.

Head and neck squamous cell carcinoma represents a group of epithelial neoplasms that exhibit considerable heterogeneity in clinical and molecular behavior [16–19]. The American Cancer Society considers the larynx as a part of the respiratory system, separated from the oral cavity and pharynx. Detailed genomic analysis of larynx cancer, performed by the Cancer Genome Atlas Research Program, demonstrated multiple potential therapeutic targets, including tyrosine kinase receptors (such as *ERbB1, ERbB2, FGFR19*), oncogenes (*CCND1, HRAS*), tumor suppressor genes (*TP53, NF1*), and the phosphoinositide 3-kinase pathway [20]. Unlike HER1 and HER2, overexpression of HER3 may be the result of increased levels of gene transcription, since no evidence of erbB3 gene amplification in head and neck tumors or other cell lines has been reported [21]. The site-dependent extreme heterogeneity of HNSCC, both from the clinical and molecular point of view [17], may contribute to differences in prognosis and to a lack of consistency in the treatment planning.

Considering that the treatment regimen could affect patient prognosis, we conducted this retrospective observational study on two series of locoregionally advanced LSCC patients treated with BioRT with cetuximab and salvage surgery or upfront surgery with or without postoperative RT/CRT, taking into account the clinical relevance and prognostic value of HER3 expression in relation to HER1 and HER2 status.

## Methods

### Patients

In this retrospective observational study, two cohorts, each consisting of 66 consecutive untreated primary advanced glottic LSCC patients (cT3-T4; unfavourable, local-extended cT2) were analyzed. One patient group was treated with BioRT with cetuximab (Group A) and the other (Group B) with upfront TL or near-total laryngectomy (NTL) with or without post-operative RT (PORT) or CRT (POCRT). Due to heterogeneity in clinical behavior of cancers arising from different larynx anatomical subsites, we excluded all patients with primary supraglottic LSCC. Both groups included patients admitted to our department of Otorhinolaryngology and Head and Neck Oncologic Unit between 1999 and 2005 (Institutional Review Head and Neck Tumor register).

Group A included patients suitable for a non-surgical organ preservation protocol according to international guidelines that were treated with cetuximab (C225) concurrently with intensity-modulated RT (IMRT), and patients cT4 any N stage refusing TL. Cetuximab was administered at an initial dose of 400 mg/m^2^ during the week before IMRT and then 250 mg/m^2^ per week during RT with a maximum of seven additional doses. All patients received dental care before the treatment. We delivered 69.96 Gy at 2.12 Gy per fraction to the planning target volume (PTV) encompassing the gross tumor volume, 59.4 Gy at 1.8 Gy per fraction to the PTV of the high-risk clinical target volume (CTV), and 54 Gy at 1.64 Gy per fraction to the PTV of the low-risk CTV. The gross tumor volumes and CTVs were each expanded 3–5 mm to generate their respective PTVs. In case of histologically proven persistent or recurrent loco-regional disease, salvage TL was performed.

Group B included patients who underwent upfront radical surgery (TL or near-TL) with or without PORT or POCRT. We performed TL in all cT3-cT4 tumors; about the selected 31 out of 66 patients with unfavourable cT2 limited-extended tumors, in 15 patients we performed a NTL (crycohyoidopexy) because of large tumor volume, deep-tissue invasion or involvement of the anterior commissure, whereas in the remaining 16 of them (including 8/16 cN+) we performed a TL due to severe vocal cord impaired mobility for deeper tissue invasion, subglottic extension, involvement of cricoarytenoid unit or posterior commissure, and poor compliance or tolerance of patients for a NTL. In case of cN+ diseases, therapeutic comprehensive radical modified neck dissection was perfomed; in case of cN0, we adopted “wait and see policy” under strict follow-up conditions. Regarding adjuvant treatment, PORT (60–70 Gy, 180 cGy per fraction) on primary tumor and neck nodal echelons with or without chemotherapy (q21 cisplatin) was administered in case of locally advanced tumors (pT4), positive (r^+^) or close resection margins, any pN+ disease.

In case of loco-regional recurrence, salvage surgery (TL or neck surgery) was performed.

All patients of both groups underwent a full diagnostic workup, including a complete head and neck evaluation, fiberoptic examination, representative biopsies, chest-CT, CT or MRI of larynx and neck. Histopathological grading was independently assessed by two pathologists, according to WHO guidelines [22]. After the workup, all cases were staged and discussed by the Tumor Board, involving at least a medical oncologist, a radiation therapist and a head and neck surgeon. Post-treatment assessments were performed 4 and 8 weeks after completion of RT or surgery. Subsequently, patients were evaluated every 3 months during the first and second years, and every 6 months during years 3 to 5. This follow-up assessment included physical examination included panendoscopy, and imaging studies consisting of computed tomography or magnetic resonance imaging of the head and neck region, and chest CT-scan.

### Immunohistochemical analysis

HER1, HER2 and HER3 expression in tumor cells was evaluated by immunohistochemistry on consecutive sections from formalin fixed and paraffin embedded tumor tissues, according to standard procedures. Tumor samples from both groups of patients were processed by the same standardized immunohistochemical procedures, utilizing anti-HER1 (clone H11, dilution 1:150; Dako, Milano, Italy), HER2 (Dilution 1:150, Dako), HER3 (RTJ.2 Dilution 1:200, DBA, Milano, Italy) monoclonal antibodies. The semi-quantitative evaluation of immunostainings was performed according to previously reported criteria [23]. Cut-off points for the expression of the HERs were chosen as previously reported [6]. Immunohistochemical assays of MCM7 and geminin proteins were performed utilizing anti-MCM7 (clone 141.2; dilution 1:100; DBA) and anti-geminin polyclonal antibody (1:200; DBA) according to the procedure previously reported [15]. Five randomly selected fields, each containing at least 400 tumor cells, were counted independently by two pathologists and labeling index for each antibody was calculated as percentage of immunostained nuclei.

### Statistical analysis

The primary endpoints went from the date of the first surgery or the beginning of bioRT with cetuximab to the date of clinical or pathological loco-regional recurrence or progression (RFS) or to the date of death (OS), regardless of the cause, or to the date of the last available information on the patient's status.

All medians and life tables were computed using the product-limit estimate by Kaplan–Meier, and the curves were examined by means of the log-rank test. Multivariate analysis was performed by Cox’s proportional hazards model. The proportional hazards assumption was assessed by visual inspection of log–log survival curves and linear regressions of scaled Schoenfeld residuals versus time. Collinearity was verified by computing variance inflation factors (VIF) from the covariance matrix of parameter estimates [24]. RFS and OS probabilities, given the covariates and follow-up time, were calculated for the model fitted by the multivariable Cox regression for both patient groups.

In order to develop a predictive model, data from both patient groups were pooled, stratifying for therapy and analyzed using Cox regression analysis. Corrected Akaike’s information criterion (AIC) was utilized in backwards stepwise procedure for selection of reduced models. Multivariable Cox proportional hazards models were developed to predict median 2- and 3-year RFS and 2.5- and 5-year OS. The performance of the final Cox models was assessed with respect to calibration and discrimination. Calibration was examined using calibration curves of the relationship between the observed survival rate and the predicted probabilities of relapse-free and overall-survival. Overfitting-corrected estimates of the performance of the final Cox models were evaluated by bootstrap with resampling with 300 repetitions, using adaptive linear spline hazard regression (25) and estimating the absolute mean error. Discrimination was evaluated by the concordance index (C-index) as the final Cox model ability to separate patient's outcomes [26].

Non-parametric Spearman’s rho was used to analyse the correlations between geminin/MCM7 ratios and predicted survivals. Kruskall-Wallis tests were used to analyse the distribution of HER phenotypes according to clinico-pathological parameters. Two-sided p < 0.05 was considered significant in statistical tests. Analyses were performed using the JMP version 13.2 (SAS Institute Inc., Cary, NC, USA), R software version 3.3.3 [27] and rms package: Regression Modeling Strategies [28].

## Results

**Fig. 1:**
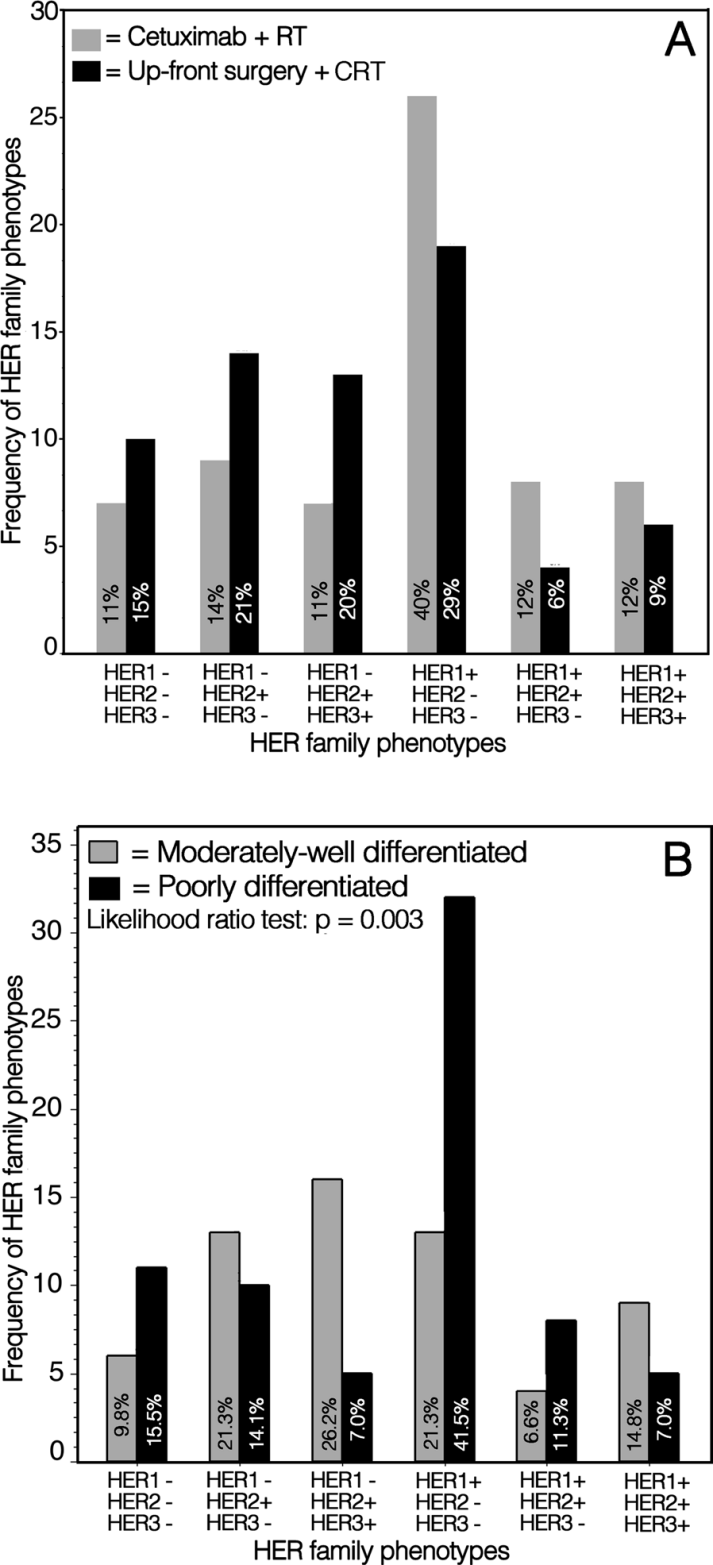
A Frequency of HER family phenotypes in LSCC of bioradiotherapy with cetuximab and in upfront surgery with or without postoperative radio/chemo-radiotherapy treated patients. Inside the bars: percentage of cases of each phenotype relative to total patient number in the group. B Frequency of HER family phenotypes in LSCC of both patient groups according to the grade of tumor differentiation

The combined expression of HER1, HER2 and HER3 and the relative frequency of the HER phenotypes were similar both in the group A and B patients (Fig. 1A). HER1^+^/HER2^−^/HER3^−^ phenotype was the most frequent and constituted 40% of tumors in the group A patients, and 29% in the group B patients, respectively. Triple positive HER1^+^/HER2^+^/HER3^+^ phenotype represented 12% and 9% of tumors in the patients of group A and B, respectively. Moreover, HER3 was expressed in both positive and negative HER1 tumors only in the presence of HER2 expression, while HER2 was also expressed independently from HER1 and HER3 (Fig. 1A).

**Fig. 2:**
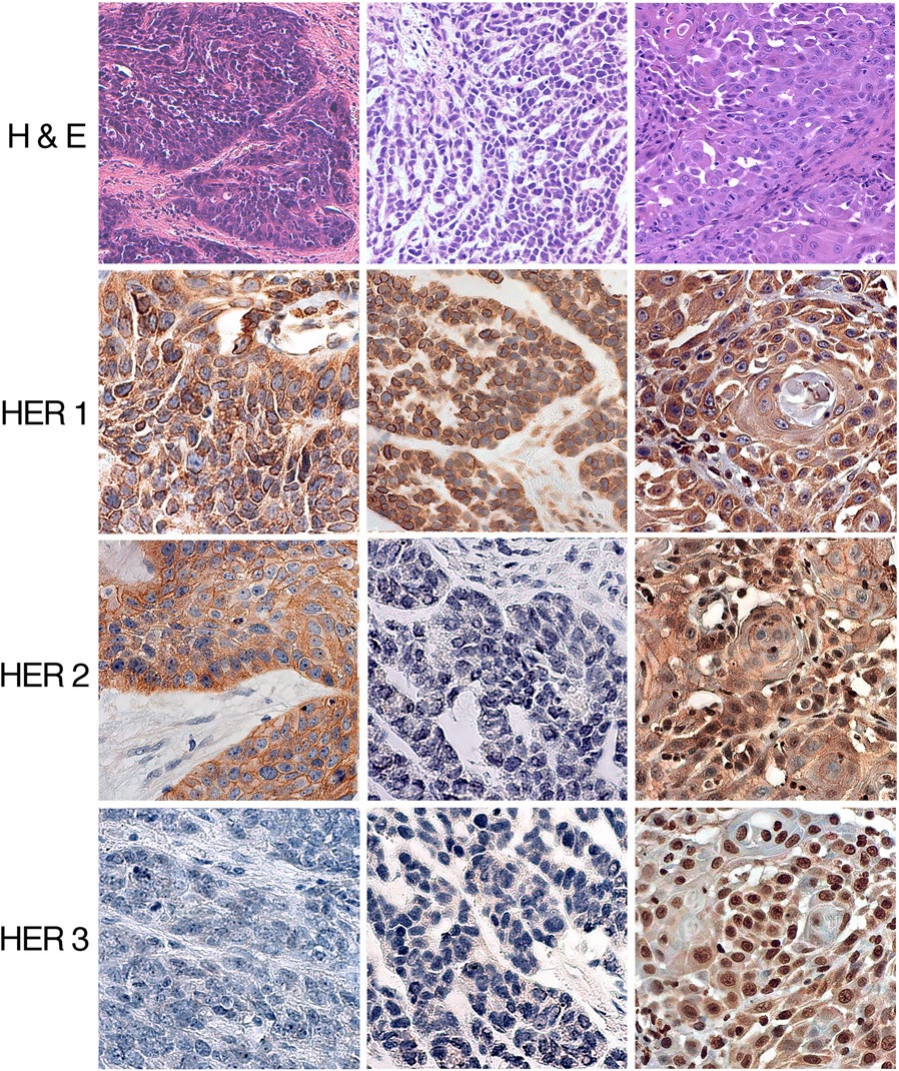
Immunohistochemical analysis of HER1, HER2 and HER3 co-expression on three LSCCs. The left and middle columns in the panel show two poorly differentiated LSCCs (H&E in the boxes above) with phenotype HER1^+^/HER2^+^/HER3^−^ and HER1^+^/HER2^−^/HER3^−^, respectively. In the right column a well differentiated LSCC (H&E in the top box) expressing the phenotype HER1^+^/HER2^+^/HER3^+^ (Original magnifications: H&E: 200×; HER1, HER2 and HER3: 400×)

Immunohistochemical labeling for HER1 and HER2 was localized both at the cell membrane and in the cytoplasm, while HER3 labeling was observed both in the cell cytoplasm and in the nucleus (Fig. 2). The HER phenotype expression patterns appeared to be associated with tumor differentiation. In particular, HER1^+^/HER2^+^/HER3^+^ phenotype was more frequently found in moderately well differentiated LSCC, while tumors not expressing HER3 were in prevalence poorly differentiated (Fig. 1B).

**Fig. 3:**
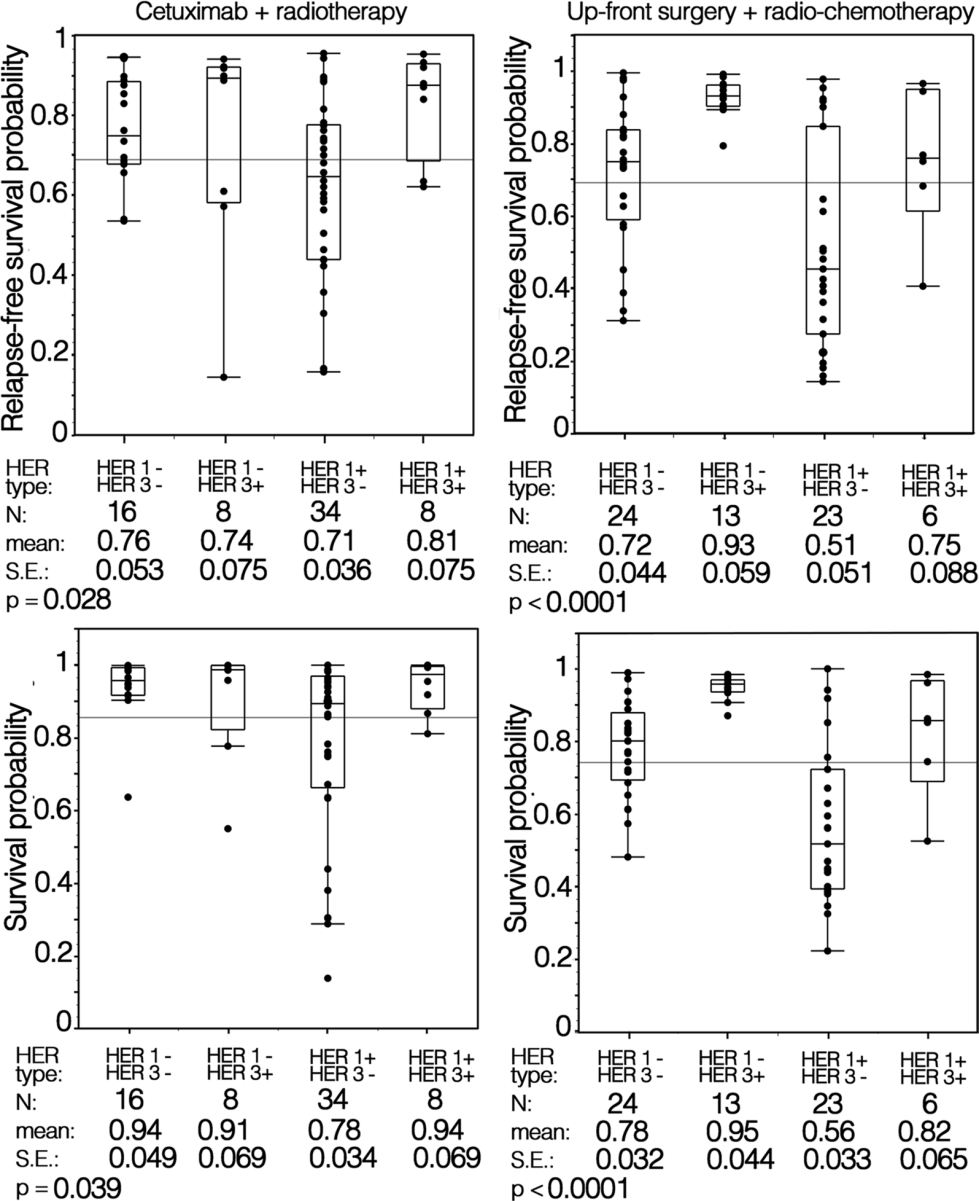
Relapse-free and overall survival probabilities according to HER phenotypes of LSCC of bioradiotherapy with cetuximab and upfront surgery with or without postoperative radio/chemo-radiotherapy treated patient groups

Multivariable analyses revealed that in both patient groups HER1 and HER3 but not HER2 expression retained independent prognostic significance relative to RFS and OS when adjusted for other clinico-pathological covariates (Table 1). In particular, HER1 positivity and HER3 tumor negativity were associated with an increased risk of relapse and death for both patients groups (Table 1). In addition, transglottic tumor site for group A patients and lymph node positivity, for group B patients behaved as independent prognostic variables of a shorter RFS. Furthermore, lymph node positivity retained prognostic significance of shorter OS only for group B patients. Based on the multivariable regression fitted model, predicted probability of relapse-free and overall survival were calculated for patients of both groups. When predicted values are grouped by HER1/HER3 co-expression status of LSCC, it clearly appears that, regardless of the therapeutic regimen, patients with HER1^+^ tumors had a reduced RFS and OS. In patients with HER1^+^ tumors, the HER3 co-expression was associated with a longer RFS and OS. Moreover, only for group B patients, the expression of HER3 was significantly associated with longer RFS and OS, regardless of HER1 co-expression in the tumors (Fig. 3).

**Table 1:** Multivariable analysis of relapse-free and overall survival in the cohorts of laryngeal squamous cancer patients treated with Cetuximab + radio-therapy (RT) or upfront surgery + radio-chemio-therapy (RCT)

Covariates:	N	Cetuximab + RT						N	Up-front surgery + RCT					
		Relapse-free survival			Overall survival				Relapse-free survival			Overall survival		
		RR	C.I. 95%	p	RR	C.I. 95%	p		RR	C.I. 95%	p	RR	C.I. 95%	p
Age (risk per year)	66	0.96	0.9–1.0	0.045	0.93	0.9–1.0	0.11	66	0.97	0.9–1.0	0.20	0.96	1.0–1.1	0.12
Site														
Glottic	47	1			1			49	1			1		
Transglottic	19	3.24	1.4–7.5	0.006	1.87	0.4–8.8	0.43	17	1.29	0.4–2.9	0.92	1.15	0.4–3.3	0.79
T														
2	36	1			1			31	1			1		
3–4	30	0.64	0.1–2.8	0.55	0.64	0.09–4.6	0.65	35	3.39	0.4–29.7	0.27	1.19	0.2–6.7	0.79
Stage														
II	29	1			1			23	1			1		
III–IV	37	1.26	0.3–6.0	0.77	11.3	0.8–165.7	0.08	43	0.30	0.03–4.0	0.30	1.03	0.1–7.1	0.98
N														
Negative	50	1			1			56	1			1		
Positive	16	0.77	0.2–2.6	0.67	1.11	0.2–5.0	0.89	10	4.35	1.5–12.8	0.008	4.18	1.2–14.2	0.022
HER-1														
Negative	24	1			1			37	1			1		
Positive	42	2.63	0.9–7.4	0.062	8.33	0.9–76.3	0.04	29	6.11	2.1–17.15	0.001	6.43	1.9–22.3	0.003
HER-2														
Negative	33	1			1			29	1			1		
Positive	33	1.04	0.4–2.7	0.94	3.44	0.5–24.0	0.21	37	1.87	0.7–5.1	0.23	1.87	0.6–5.9	0.28
HER-3														
Negative	50	1			1			47	1			1		
Positive	16	0.17	0.04–0.69	0.013	0.03	0.002–0.5	0.012	19	0.15	0.04–0.65	0.010	0.13	0.02–0.7	0.019
Concordance index		0.77			0.83				0.78			0.78		

*RR* Reference risk

*C.I 95%* Conficence interval 95%

*p* p-value likelihood ratio test

**Fig. 4:**
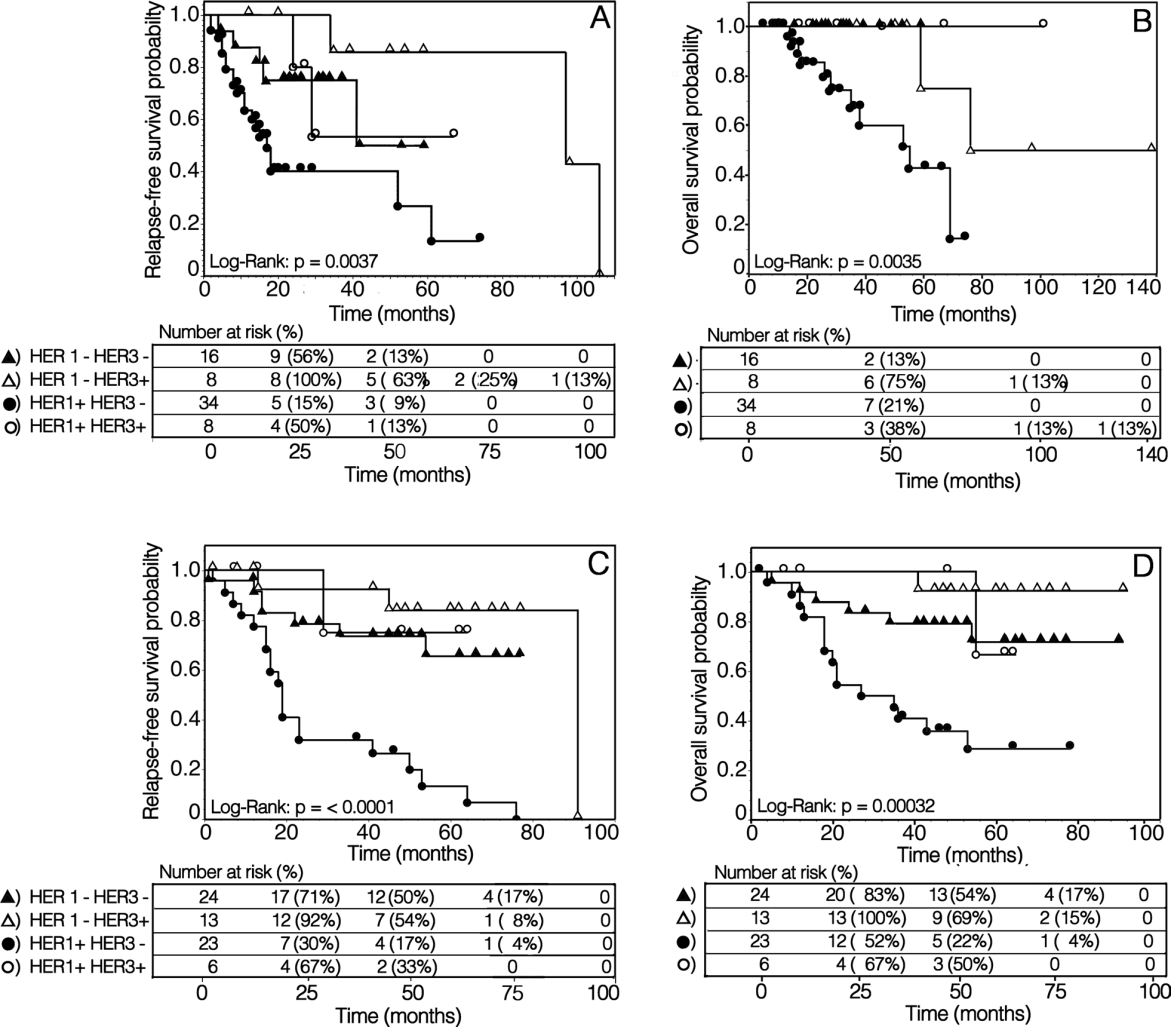
Kaplan–Meier analyses of survival curves as a function of HER family phenotypes in LSCC. Bioradiotherapy with cetuximab (A, B) and upfront surgery with or without postoperative radio/chemo-radiotherapy (C, D) treated patients

**Fig. 5:**
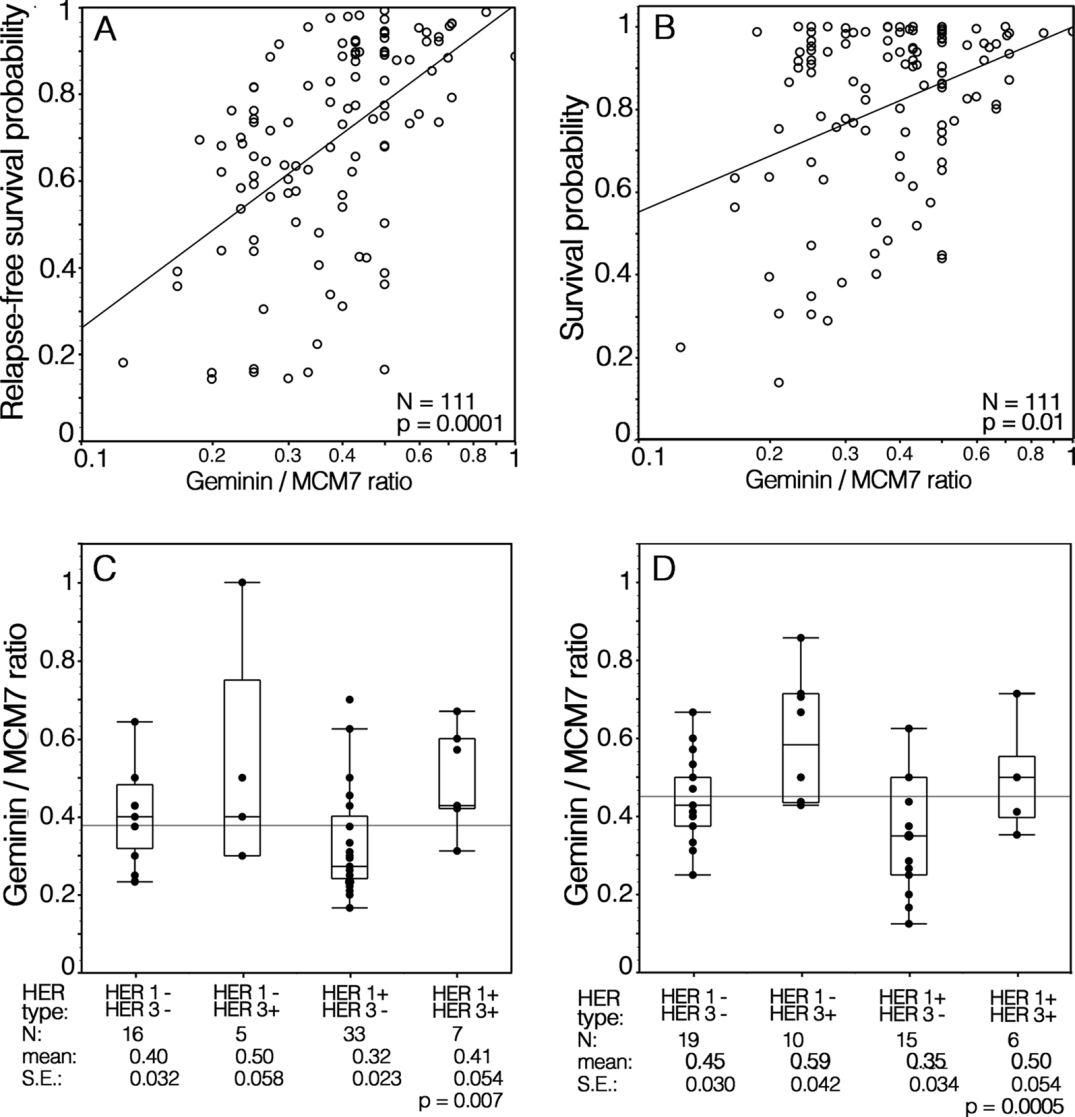
Plots of the relapse-free (A) and overall (B) survival probabilities predicted by the multivariable Cox’s regression fitted model as a function of the combined geminin/MCM7 labeling indices (LI) from LSCC of both patient groups. The ratios of geminin/MCM7 LI according to HER1/HER3 phenotypes of LSCC of bioradiotherapy with cetuximab (C) and upfront surgery with or without postoperative radio/chemo-radiotherapy (D) treated patient groups

Kaplan–Meier analysis of survival curves, according to HER1/HER3 phenotype of LSCC, confirmed that in both patient cohorts the expression of HER1 was associated with reduced RFS and OS. Moreover, in HER1^+^ tumors, the co-expression of HER3 was significantly associated with prolonged RFS and OS (Fig. 4). In both patient groups, the gemin/MCM7 ratio, utilized as an estimate of the proliferating cell fraction in the S-G2-M phase of the cell cycle, showed a significant positive correlation with the RFS and OS predicted by the multivariable Cox’s regression fitted model (Fig. 5A, B).

Furthermore, when the geminin/MCM7 ratios were grouped by HER phenotypes, it was evident that tumors expressing HER3 had a higher geminin/MCM7 ratio than HER3 negative ones, regardless of HER1 co-expression (Fig. 5C, D). On the other hand, the geminin labeling index was significantly higher in HER3^+^ than in HER3^−^ tumors (23.88 ± 1.09 SE versus 35.54 ± 1.89 SE; p < 0.0001).

In order to develop predictive models, we pooled data from the two patient series and stratified them by type of therapy, considering that in both patient groups HER1 and HER3 had prognostic significance independent of the type of therapy.

Multivariable Cox proportional hazards regression analyses of the two grouped patient’s series confirmed that HER1 and HER3 retained an independent prognostic significance and that the model had an apparent good accuracy with a C-index of 0.77 and 0.81 for RFS and OS, respectively (data not shown). After model selection, we obtained reduced models by backwards stepwise procedure. Beta coefficients and hazard ratios of variables are listed in the Additional file 1: Figure S1.

The models were internally validated with respect to calibration and discrimination. Discrimination suggested a good accuracy with a bootstrap-corrected C-indices of 0.76 and 0.79 for RFS and OS, respectively. The closeness of the calibration curves for RFS and OS to the ideal 45° calibration lines suggests that the models are well calibrated for predictions on an absolute probability scale (Additional file 2: Figure S2). The absolute values of the differences between the predicted value and the observed value (Mean Absolute Error: MAE) were 0.04 and 0.02 for RFS and OS, respecively. Moreover, the optimism-corrected slope shrinkage (0.90 and 0.93 for RFS and OS, respectively) indicates little overfitting.

## Discussion

HER family member receptors homo- or hetero-dimerize, activating multiple signaling pathways, which guide subsequent cell behaviors. Expression in cancer cells of more than one HER receptor complicates the understanding of how specific homo- or hetero-dimer contributes to cell behavior and potential malignant trasformation. In order to include biomarkers in guidelines for selecting patients for specific treatments, it is necessary to distinguish the prognostic effects of these factors from their ability to predict a differential clinical benefit from the specific treatment, considering that protocol regimen inevitably affects patient prognosis.

This study, conducted on two patient groups receiving different treatments, offers insight on the relevance of different patterns of coexpression of HER1, HER2 and HER3 on outcome and therapy response. Our data showed that the most frequent tumor phenotype was represented by HER1^+^/HER2^−^/HER3^−^, and it was more frequently detected in the subgroup of poorly differentiated LSCC, characterized by the shortest RFS and OS, independently from the treatment protocols. This finding is in accord with the well-known worse prognostic significance of HER1 expression in LSCC, particularly when HER1 expression is evaluated by quantitative [29, 30] or semi-quantitative immunohistochemistry [6, 31]. Interestingly, patients with HER1^+^ tumors, treated with upfront surgery with or without postoperative RT/CRT, showed a greater risk of relapse and death than patients treated with BioRT with cetuximab. It is possible that cetuximab may sensitize to therapeutic agents tumors with a high HER1 expression, thus reducing the prognostic significance of HER1 status. In accord with this hypothesis are previous observations in HNSCC, showing that a high HER1 tumor expression predicted a worse clinical outcome in patients treated with radiation alone, while it did not reveal the same predictive significance in patients treated with radiation-cisplatin plus cetuximab, although assessed with the same method [4, 32].

According to previous observations [33–35], in both patient groups, we found that HER2 did not behave as an independent prognostic marker of RFS and OS. In HNSCC it has been previously found that HER2 is co-expressed with HER1 [36], and this co-expression may contribute to the negative prognostic impact of HER1, being associated with resistance to therapeutic agents [37, 38]. Moreover, we found, in both patient groups, that HER1 and HER2 were prevalently co-expressed in poorly differentiated tumors with short RFS and OS. In addition, HER3 was expressed only in combination with HER2, in accordance with the known impaired kinase activity of HER3 and its preferred hetero-dimerization with HER2 [39, 40]. In both patient groups, a high HER1/HER3 co-expression correlated with a better prognosis, as compared to the high expression of HER1 without co-expression of HER3.

In addition to be a target of the clinical development of anti-HER3 therapies, the predictive and prognostic role of HER3 over-expression in malignant solid tumors is also the focus of clinical attention, although the research findings are contradictory. In fact, contrasting results concerning the prognostic value of HER3 expression in different cancer types have been reported in the literature [41]. In addition to the differences in clinical staging, sample sizes and types of antibodies used for immunohistochemistry, the inherent diversity and complexity of each tumor type may have influenced the results found in the absence of stratified analyses. In this regard, for example, the prognostic role of HER3 expression on survival is negative for gastric cancer, while it is uncertain for breast and colon cancers [41]. From our results, HER3 positivity was found in 29% of group A and 24% of group B LSCC patients, respectively. These data are in accord with previous observations in larynx cancer [42].

Notheworthy, our findings that HER3 expression was most frequently associated with well differentiated and rarely with poorly differentiated carcinomas, are in accord with the data of Wei et al. showing that basaloid squamous cell carcinoma of the larynx does not express HER3 [42]. HER3 expression in more differentiated tumor histotypes has been found also in breast [43], colon [44] and bladder cancer [45].

Takikita et al. [11] in HNSCC reported that HER3 can be detected with either a cytoplasmic or a membranous prevalent expression pattern and that only membranous expression was significantly associated with worse overall survival. However, our present study on LSCC, in accord to previous observations [6, 46], shows that HER3 expressed at both cytoplasmic and nuclear level, prevalently in moderately to well differentiated histotypes, is associated with a better survival. It is possible that the heterogeneity of tumors included in HNSCC, either from anatomical, clinical and molecular aspects [17], compared to the homogeneity of the larynx cancers analyzed in our study, could have contributed to differences in the pattern of HER3 expression and their prognostic significance. In fact, unlike what happens in cell lines, at the tissue level the co-expression of HER family receptors can be dissimilar in different cell domains within the context of the tissue architecture of the tumors. In this respect, complex 3D cultures are more predictive of the clinical outcome than their 2D counterparts as protein expression and kinase activities of ErbB family members were substantially altered in the 3D cancer models as compared to 2D ones [47]. Interestingly, in well differentiated LSCC we found that HER3 was mainly expressed in spinous and supra-basal layers, while HER1 was mainly expressed by cells of the basal layer [46], similarly to normal epidermis. These findings are also consistent with observations in vitro, where differentiation of MTSV1-7 breast cells was accompanied by increasing concentration of HER3 in the nucleoli [48].

Surprisingly, although HER3 is known as a transmembrane protein, we did not observe HER3 labeling at the cell membrane level. The immunostaining pattern of HER3 reported by different authors in different cancers is not univocal and variable localizations have been reported, such as cytoplasmic [49, 50], membranous and cytoplasmic [44, 51], restricted to the nucleus [52, 53] or nuclear and cytoplasmic [6, 46].

The finding of different subcellular HER3 localizations may be linked to inherent peculiarities of a single tumor. However, other explanations for some of the reported differences in HER3 immunolocalization could rest on the use of different antibodies, since it has been reported that only a few monoclonal antibodies can label HER3 localized in the nucleus [48]. In addition, the possibility that some immunostaining at the membrane level has been masked by the pronounced cytoplasmic positivity cannot be excluded. Previous studies showed that HER receptor proteins can translocate from the membrane to the nucleus in a variety of cancer cells [48, 52, 54, 55] and that this event may play an important role in cancer biology.

In this respect, it is interesting the finding that nuclear and cytoplasmic patterns of HER4 expression were present in different areas of the same LSCC and that the prevalence of nuclear pattern in the tumors was associated with a longer patient’s survival [6]. This finding further suggests that the analysis of sub-cellular expression of HER family receptors may be important to understand the characteristics of the biological behavior of the tumor.

We found that HER3 expression acts as a prognostic marker in LSCC as its expression correlated with a better prognosis, independently from therapeutic regimen. This result is in contrast with several lines of preclinical evidence that HER3 signaling may be a critical pathway for acquired resistance to cetuximab. Despite preclinical data strongly supported a potential role of HER3 signaling in acquired resistance to HER1 inhibitors [56], two randomized phase II study in HNSCC [53] and metastatic colorectal cancers [57] showed no benefits of the dual inhibition of HER1 and HER3 with duligotuzumab as compared to inhibition of HER1 alone with cetuximab. These findings suggest that inhibition of HER1 alone is sufficient to block HER1/HER3 signaling, being minimal the role of HER2 in these cancer types. According to this suggestion, we found that HER1 and HER3, but not HER2, behave as independent prognostic markers in LSCC. However, the role of dual HER1/HER3 inhibition to overcome resistance to therapy remains not well understood in patients not previously treated with HER inhibitors.

Interestingly, we found that expression of HER3 was a favourable prognostic marker also in patients treated with upfront surgery with or without postoperative RT/CRT. This finding suggests that HER3 expression behaves as a prognostic marker independent from cetuximab treatment in LSCC. Considering the prominent nuclear localization of HER3 in princkle-like suprabasal squamous cancer cells of well differentiated LSCC, it is possible that HER3 nuclear localization prevents its interaction with other HER family members at the level of the cytoplasmic membrane and the consequent activation of downstream signaling pathways. This possibility could also explain the previous findings in HNSCC that the single inhibition of HER1 with cetuximab has an efficacy similar to the dual HER1/HER3 inhibition with duligotuzumab [58].

It is known that geminin is expressed during S, G2, and early M phases of the cell cycle [59] and high geminin/MCM7 ratio might reflects proliferating tumors in S-G2-M phase of the cell cycle. HER1^+^/HER3^+^ LSCC had a higher percentage of geminin positive cells than HER1^+^/HER3^−^ ones, suggesting an increased proliferation rate of cancer cells when HER3 was co-expressed. Moreover, HER3^+^ expressing tumors showed a higher geminin labeling index as compared to HER3^−^ ones.

It has been reported that nuclear HER3 is a transcriptional co-activator of cyclin D1 promoter by its C-terminal transactivation domain, stimulating cell transition from G1 to S phase of the cell cycle [60]. Moreover, in patients treated with BioRT with cetuximab or upfront surgery with or without postoperative RT/CRT, LSCC with HER1^+^/HER3^+^ phenotype and high geminin/MCM7 ratio were associated with a better relapse-free and overall survival predicted probability. Considering that both patient cohorts were treated with RT or CRT and that geminin might reflect proliferating cells in S-G2-M phase, it is possible that tumors co-expressing HER3 could be more susceptible to chemo–radio treatment. This possibility is also supported by previous obervations that in high-grade astrocytic brain tumors [61], rectal cancer [58], oral squamous cell carcinoma [62] and LSCC [15] high expression of geminin is associated with a more favorable prognosis in patients receiving chemo-radiotherapy.

Based on our findings, we hypothesize that the nuclear sequestering of ErbB3 receptors may alter the propensity to form ErbB3-containing homodimers or heterodimers at the level of the plasma membrane. This could alter the response to growth factors that favor pathways that are activated by ErbB family members that remain in the cytoplasmic membrane. Interestingly, it has been observed that clathrin-dependent endocytosis pathway regulates cytoplasmic trafficking, whereas nuclear translocation of ERBB3 relies on the high-capacity clathrin-independent pathway, thereby allowing ERBB3 regulated cytoplasmic signaling to be modified independent of nuclear signaling (63). In the light of our findings, it appears of extreme importance to understand which are the signaling pathways that favor the nuclear translocation of HER3. Further work will be needed on in vivo and in vitro tumor cell models to evaluate these possibilities in the case of LSCC.

## Conclusions

As we move from translational research forward into an era of precision medicine, we must tailor the treatment to the specific intrinsic biological behavior of the tumor for each patient, particularly regarding the sensitivity of tumor cells to cetuximab and to radiation, in order to achieve a balance between overall survival, larynx preservation and quality of life.

In this study, we developed and validated a simple prognostic model based on nuclear HER3 expression that allows to improve the prognostic stratification of patients with HER1 positive advanced LSCC. This prognostic model could help clinicians to identify a subgroup of patients who do not need to be treated with double inhibition with anti-HER1 and anti-HER3, but can benefit from BioRT with cetuximab alone.

This study had some limitations. First, this model was derived from retrospective data, making it susceptible to a data collection bias. Second, the portability of this model to other cohorts needs to be externally validated.

## Supplementary Information

Additional file 1: Figure S1. Forest plot of the relative estimates and hazard ratio of covariates for relapse-free and overall survival.

Additional file 2: Figure S2. Plots of bootstrap estimates of calibration accuracy for the indicated month estimates from the Cox models, using adaptive linear spline hazard regression. The gray scale line is the line of identity of observed-predicted relationship, representing the ideal calibration curve; the smooth black curve is the apparent calibration estimated by linear spline hazard regression; the blue line is the bootstrap overfitting-corrected calibration curve estimated also by hazard regression. Mean Absolute Error (MAE) is the mean of the absolute errors. The absolute error is the absolute value of the difference between the predicted value and the observed value.

## Data Availability

Derived data supporting the findings of this study are available from the corresponding author on request.

## Abbreviations

BioRT: Bio-radiotherapy
CRT: Chemoradiotherapy
CT: Chemotherapy
CTV: Clinical target volume
EGFR: Epidermal growth factor receptor
HER: Human epidermal growth factor family receptors
HNSCC: Head and neck squamous cell carcinoma
IMRT: Intensity-modulated radiotherapy
LSCC: Laryngeal squamous cell carcinoma
MCM: Minichromosome maintenance
NTL: Near-total laryngectomy
OS: Overall survival
PORT: Post-operative radiotherapy
POCRT: Post-operative chemoradiotherapy
PTV: Planning target volume
RFS: Relapse-free survival
RT: Radiotherapy
TL: Total laryngectomy

## References

[1] Siegel RL, Miller KD, Jemal A (2019). Cancer statistics, 2019. CA Cancer J Clin.

[2] Bonner JA, Harari PM, Giralt J, Azarnia N, Shin DM, Cohen RB (2006). Radiotherapy plus cetuximab for squamous-cell carcinoma of the head and neck. N Engl J Med.

[3] Bonner J, Giralt J, Harari P, Spencer S, Schulten J, Hossain A, et al. Cetuximab and Radiotherapy in laryngeal preservation for cancers of the larynx and hypopharynx: a secondary analysis of a randomized clinical trial. JAMA Otolaryngol Head Neck Surg. JAMA Otolaryngol Head Neck Surg. 2016;142:842–9. Erratum in: JAMA Otolaryngol Head Neck Surg. 2017;143:97. Erratum in: JAMA Otolaryngol Head Neck Surg. 2019;145:96. 10.1001/jamaoto.2016.1228 PMC5025385 27389475

[4] Ang KK, Zhang Q, Rosenthal DI, Nguyen-Tan PF, Sherman EJ, Weber RS (2014). Randomized phase III trial of concurrent accelerated radiation plus cisplatin with or without cetuximab for stage III to IV head and neck carcinoma: RTOG 0522. J Clin Oncol.

[5] Chong CR, Jänne PA (2013). The quest to overcome resistance to EGFR-targeted therapies in cancer. Nat Med.

[6] Almadori G, Bussu F, Gessi M, Ferrandina G, Scambia G, Lauriola L (2010). Prognostic significance and clinical relevance of the expression of the HER family of type I receptor tyrosine kinases in human laryngeal squamous cell carcinoma. Eur J Cancer.

[7] Olayioye MA, Neve RM, Lane HA, Hynes NE (2000). The ErbB signaling network: receptor heterodimerization in development and cancer. EMBO J.

[8] Xia W, Lau YK, Zhang HZ, Xiao FY, Johnston DA, Liu AR (1999). Combination of EGFR, HER-2/neu, and HER-3 is a stronger predictor for the outcome of oral squamous cell carcinoma than any individual family members. Clin Cancer Res.

[9] Sakurai K, Urade M, Takahashi Y, Kishimoto H, Noguchi K, Yasoshima H (2000). Increased expression of c-erbB-3 protein and proliferating cell nuclear antigen during development of verrucous carcinoma of the oral mucosa. Cancer.

[10] Shintani S, Funayama T, Yoshihama Y, Alcalde RE, Matsumura T (1995). Prognostic significance of ERBB3 overexpression in oral squamous cell carcinoma. Cancer Lett.

[11] Takikita M, Xie R, Chung JY, Cho H, Ylaya K, Hong SM (2011). Membranous expression of Her3 is associated with a decreased survival in head and neck squamous cell carcinoma. J Transl Med.

[12] De Vicente JC, Esteban I, Germanà P, Germanà A, Vega JA (2003). Expression of ErbB-3 and ErbB-4 protooncogene proteins in oral squamous cell carcinoma: a pilot study. Med Oral.

[13] Tachibana KE, Gonzalez MA, Coleman N (2005). Cell-cycle-dependent regulation of DNA replication and its relevance to cancer pathology. J Pathol.

[14] Montanari M, Boninsegna A, Faraglia B, Coco C, Giordano A, Cittadini A (2005). Increased expression of geminin stimulates the growth of mammary epithelial cells and is a frequent event in human tumors. J Cell Physiol.

[15] Almadori G, Lauriola L, Coli A, Bussu F, Gallus R, Scannone D (2017). Minichromosome maintenance protein 7 and geminin expression: Prognostic value in laryngeal squamous cell carcinoma in patients treated with radiotherapy and cetuximab. Head Neck.

[16] Huang Q, Yu GP, McCormick SA, Mo J, Datta B, Mahimkar M (2002). Genetic differences detected by comparative genomic hybridization in head and neck squamous cell carcinomas from different tumor sites: construction of oncogenetic trees for tumor progression. Genes Chromosomes Cancer.

[17] Bosch FX, Ritter D, Enders C, Flechtenmacher C, Abel U, Dietz A (2004). Head and neck tumor sites differ in prevalence and spectrum of p53 alterations but these have limited prognostic value. Int J Cancer.

[18] Patel SG, Shah JP (2005). TNM staging of cancers of the head and neck: striving for uniformity among diversity. CA Cancer J Clin.

[19] Mroz EA, Tward AD, Pickering CR, Myers JN, Ferris RL, Rocco JW (2013). High intratumor genetic heterogeneity is related to worse outcome in patients with head and neck squamous cell carcinoma. Cancer.

[20] Steuer CE, El-Deiry M, Parks JR, Higgins KA, Saba NF (2017). An update on larynx cancer. CA Cancer J Clin.

[21] Issing WJ, Heppt WJ, Kastenbauer ER (1993). erbB-3, a third member of the erbB/epidermal growth factor receptor gene family: its expression in head and neck cancer cell lines. Eur Arch Otorhinolaryngol.

[22] El-Naggar AK, Chan JKC, Grandis JR, Takata T, Slootweg PJ, editors. World Health Organization Classification of Head and Neck Tumours. IARC, Lyon, 2017. 10.1016/j.humpath.2017.05.014 28583885

[23] Ranelletti FO, Almadori G, Rocca B, Ferrandina G, Ciabattoni G, Habib A (2001). Prognostic significance of cyclooxygenase-2 in laryngeal squamous cell carcinoma. Int J Cancer.

[24] Davis CE, Hyde JE, Bangdiwala SI, Nelson JJ, Moolgavkar SH, Prentice RL (1986). An example of dependencies among variables in a conditional logistic regression. Modern statistical methods in chronic disease epidemiology.

[25] Kooperberg C, Stone CJ, Truong YK (1995). Hazard regression. J Am Stat Assoc.

[26] Harrell FE. Regression Modeling Strategies. With applications to linear models, logistic and ordinal regression, and survival analysis. 2nd edn. Springer Series in Statistics, Springer International Publishing, Springer Nature Switzerland AG, 2015. ISBN 978-3-319-19424-0. 10.1007/978-3-319-19425-7.

[27] R Development Core Team: A language and environment for statistical computing. R Foundation for Statistical Computing Vienna, AT, 2011. https://www.Rproject.org/.

[28] Harrell FE Jr (2016). RMS: regression modeling strategies. R package version 4.5-0. http://CRAN.R-project.org/package=rms.

[29] Maurizi M, Scambia G, Panici PB (1992). EGF receptor expression in primary laryngeal cancer: correlation with clinico-pathological features and prognostic significance. Int J Cancer.

[30] Rubin Grandis J, Melhem MF, Gooding WE, Day R, Holst VA, Wagener MM (1998). Levels of TGF-alpha and EGFR protein in head and neck squamous cell carcinoma and patient survival. J Natl Cancer Inst.

[31] Wen QH, Miwa T, Yoshizaki T, Nagayama I, Furukawa M, Nishijima H (1996). Prognostic value of EGFR and TGF-alpha in early laryngeal cancer treated with radiotherapy. Laryngoscope.

[32] Chung CH, Zhang Q, Hammond EM, Trotti AM, Wang H, Spencer S (2011). Integrating epidermal growth factor receptor assay with clinical parameters improves risk classification for relapse and survival in head-and-neck squamous cell carcinoma. Int J Radiat Oncol Biol Phys.

[33] Pollock NI, Grandis JR (2015). HER2 as a therapeutic target in head and neck squamous cell carcinoma. Clin Cancer Res.

[34] Ali MA, Gunduz M, Gunduz E, Tamamura R, Beder LB, Katase N (2010). Expression and mutation analysis of her2 in head and neck squamous cell carcinoma. Cancer Invest.

[35] Freier K, Joos S, Flechtenmacher C, Devens F, Benner A, Bosch FX (2003). Tissue microarray analysis reveals site-specific prevalence of oncogene amplifications in head and neck squamous cell carcinoma. Cancer Res.

[36] Ibrahim SO, Lillehaug JR, Johannessen AC, Liavaag PG, Nilsen R, Vasstrand EN (1999). Expression of biomarkers (p53, transforming growth factor alpha, epidermal growth factor receptor, c-erbB-2/neu and the proliferative cell nuclear antigen) in oropharyngeal squamous cell carcinomas. Oral Oncol.

[37] Quesnelle KM, Grandis JR (2011). Dual kinase inhibition of EGFR and HER2 overcomes resistance to cetuximab in a novel in vivo model of acquired cetuximab resistance. Clin Cancer Res.

[38] Schneider MR, Yarden Y (2016). The EGFR-HER2 module: a stem cell approach to understanding a prime target and driver of solid tumors. Oncogene.

[39] Tzahar E, Waterman H, Chen X, Levkowitz G, Karunagaran D, Lavi S (1996). A hierarchical network of interreceptor interactions determines signal transduction by Neu differentiation factor/neuregulin and epidermal growth factor. Mol Cell Biol.

[40] Yarden Y, Pines G (2012). The ERBB network: at last, cancer therapy meets systems biology. Nat Rev Cancer.

[41] Li Q, Zhang R, Yan H, Zhao P, Wu L, Wang H (2017). Prognostic significance of HER3 in patients with malignant solid tumors. Oncotarget.

[42] Wei Q, Sheng L, Shui Y, Hu Q, Nordgren H, Carlsson J (2008). EGFR, HER2, and HER3 expression in laryngeal primary tumors and corresponding metastases. Ann Surg Oncol.

[43] Lee Y, Cho S, Seo JH, Shin BK, Kim HK, Kim I (2007). Correlated expression of erbB-3 with hormone receptor expression and favorable clinical outcome in invasive ductal carcinomas of the breast. Am J Clin Pathol.

[44] Seo AN, Kwak Y, Kim WH, Kim DW, Kang SB, Choe G (2015). HER3 protein expression in relation to HER2 positivity in patients with primary colorectal cancer: clinical relevance and prognostic value. Virchows Arch.

[45] Memon AA, Sorensen BS, Meldgaard P, Fokdal L, Thykjaer T, Nexo E (2006). The relation between survival and expression of HER1 and HER2 depends on the expression of HER3 and HER4: a study in bladder cancer patients. Br J Cancer.

[46] Bussu F, Ranelletti FO, Gessi M, Graziani C, Lanza P, Lauriola L (2012). Immunohistochemical expression patterns of the HER4 receptors in normal mucosa and in laryngeal squamous cell carcinomas: antioncogenic significance of the HER4 protein in laryngeal squamous cell carcinoma. Laryngoscope.

[47] Jacobi N, Seeboeck R, Hofmann E, Schweiger H, Smolinska V, Mohr T (2017). Organotypic three-dimensional cancer cell cultures mirror drug responses in vivo: lessons learned from the inhibition of EGFR signaling. Oncotarget.

[48] Offterdinger M, Schöfer C, Weipoltshammer K, Grunt TW (2002). c-erbB-3: a nuclear protein in mammary epithelial cells. J Cell Biol.

[49] Wei Q, Chen L, Sheng L, Nordgren H, Wester K, Carlsson J (2007). EGFR, HER2 and HER3 expression in esophageal primary tumours and corresponding metastases. Int J Oncol.

[50] Unger U, Denkert C, Braicu I, Sehouli J, Dietel M, Loibl S (2017). Prognostic impact of HER3 based on protein and mRNA expression in high-grade serous ovarian carcinoma. Virchows Arch.

[51] Hayashi M, Inokuchi M, Takagi Y, Yamada H, Kojima K, Kumagai J (2008). High expression of HER3 is associated with a decreased survival in gastric cancer. Clin Cancer Res.

[52] Koumakpayi IH, Diallo JS, Le Page C, Lessard L, Gleave M, Bégin LR (2006). Expression and nuclear localization of ErbB3 in prostate cancer. Clin Cancer Res.

[53] Trocmé E, Mougiakakos D, Johansson CC, All-Eriksson C, Economou MA, Larsson O (2012). Nuclear HER3 is associated with favorable overall survival in uveal melanoma. Int J Cancer.

[54] Lin SY, Makino K, Xia W, Matin A, Wen Y, Kwong KY (2001). Nuclear localization of EGF receptor and its potential new role as a transcription factor. Nat Cell Biol.

[55] Wang SC, Lien HC, Xia W, Chen IF, Lo HW, Wang Z (2004). Binding at and transactivation of the COX-2 promoter by nuclear tyrosine kinase receptor ErbB-2. Cancer Cell.

[56] Huang S, Li C, Armstrong EA, Peet CR, Saker J, Amler LC (2013). Dual targeting of EGFR and HER3 with MEHD7945A overcomes acquired resistance to EGFR inhibitors and radiation. Cancer Res.

[57] Hill AG, Findlay MP, Burge ME, Jackson C, Alfonso PG, Samuel L (2018). Phase II study of the dual EGFR/HER3 inhibitor duligotuzumab (MEHD7945A) versus cetuximab in combination with FOLFIRI in second-Line *RAS* wild-type metastatic colorectal cancer. Clin Cancer Res.

[58] Fayette J, Wirth L, Oprean C, Udrea A, Jimeno A, Rischin D (2016). Randomized phase II study of duligotuzumab (MEHD7945A) vs. cetuximab in squamous cell carcinoma of the head and neck (MEHGAN Study). Front Oncol.

[59] McGarry TJ, Kirschner MW (1998). Geminin, an inhibitor of DNA replication, is degraded during mitosis. Cell.

[60] Brand TM, Iida M, Luthar N, Wleklinski MJ, Starr MM, Wheeler DL (2013). Mapping C-terminal transactivation domains of the nuclear HER family receptor tyrosine kinase HER3. PLoS ONE.

[61] Shrestha P, Saito T, Hama S, Arifin MT, Kajiwara Y, Yamasaki F (2007). Geminin: a good prognostic factor in high-grade astrocytic brain tumors. Cancer.

[62] Tamura T, Shomori K, Haruki T, Nosaka K, Hamamoto Y, Shiomi T (2010). Minichromosome maintenance-7 and geminin are reliable prognostic markers in patients with oral squamous cell carcinoma: immunohistochemical study. J Oral Pathol Med.

[63] Reif R, Adawy A, Vartak N, Schröder J, Günther G, Ghallab A (2016). Activated ErbB3 translocates to the nucleus via clathrin-independent endocytosis, which is associated with proliferating cells. J Biol Chem.

