# Supplementary figures and images for: Nuclear HER3 expression improves the prognostic stratification of patients with HER1 positive advanced laryngeal squamous cell carcinoma
Source: J Transl Med. 2021 Sep 27;19:408. doi: 10.1186/s12967-021-03081-0 (PMC8477517; doi:10.1186/s12967-021-03081-0)

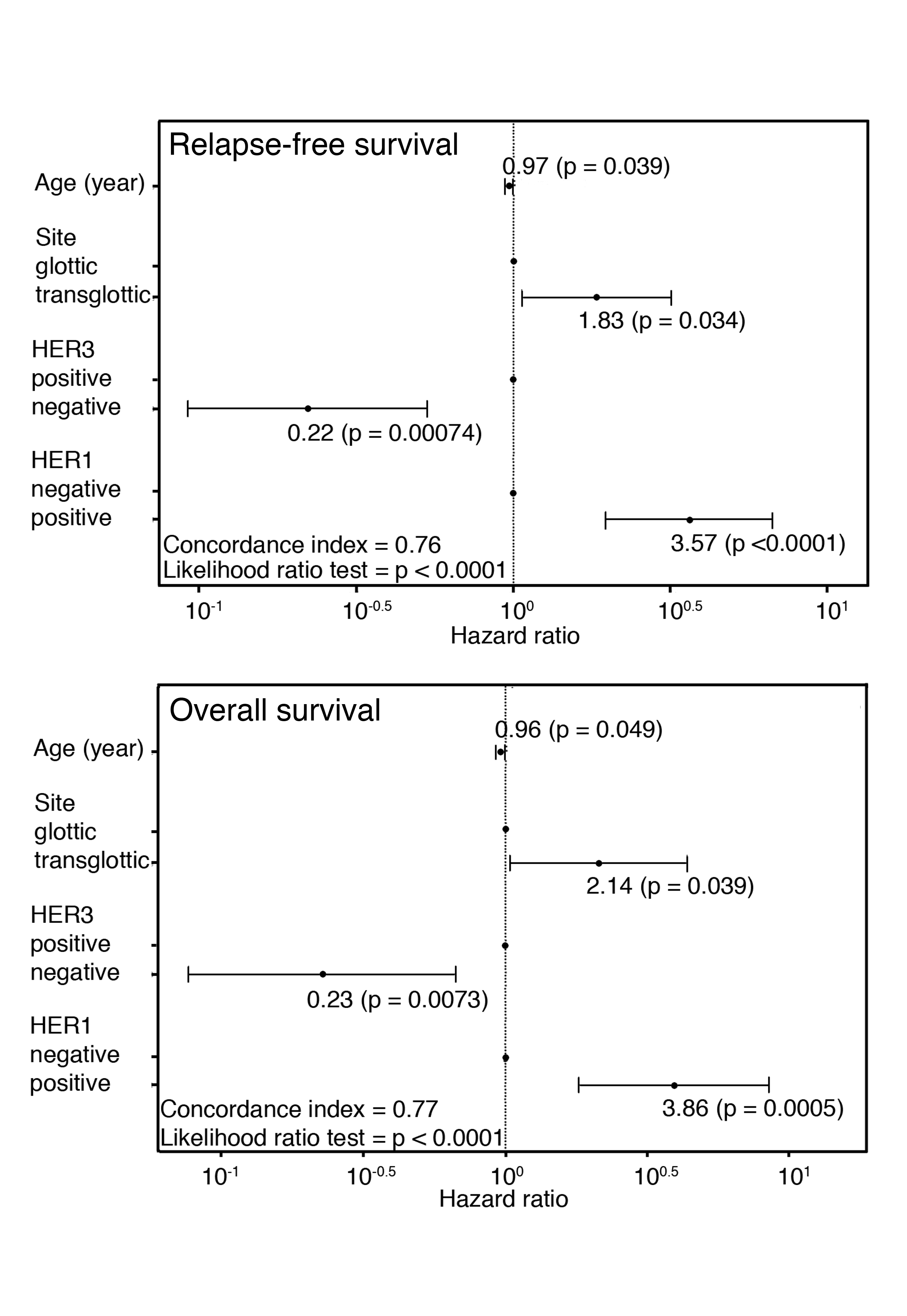

Supplement: Supplementary file 1 — Additional file 1: Figure S1. Forest plot of the relative estimates and hazard ratio of covariates for relapse-free and overall survival. [file 12967_2021_3081_MOESM1_ESM.tif]

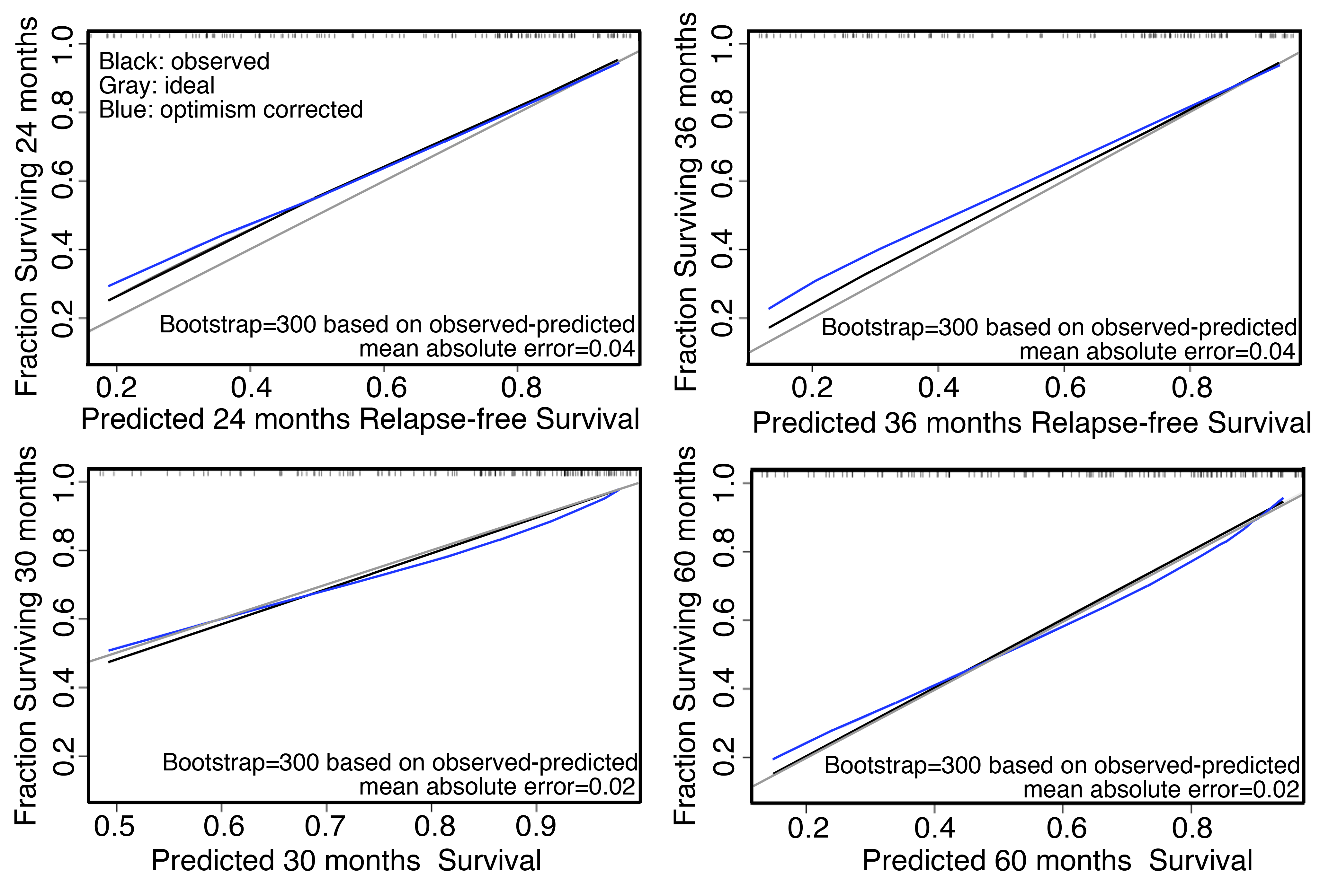

Supplement: Supplementary file 2 — Additional file 2: Figure S2. Plots of bootstrap estimates of calibration accuracy for the indicated month estimates from the Cox models, using adaptive linear spline hazard regression. The gray scale line is the line of identity of observed-predicted relationship, representing the ideal calibration curve; the smooth black curve is the apparent calibration estimated by linear spline hazard regression; the blue line is the bootstrap overfitting-corrected calibration curve estimated also by hazard regression. Mean Absolute Error (MAE) is the mean of the absolute errors. The absolute error is the absolute value of the difference between the predicted value and the observed value. [file 12967_2021_3081_MOESM2_ESM.tif]
